# Point-of-care Ultrasound Training During an Emergency Medicine Clerkship: A Prospective Study

**DOI:** 10.7759/cureus.6118

**Published:** 2019-11-10

**Authors:** Arif Alper Cevik, Elif Dilek Cakal, Fikri Abu-Zidan

**Affiliations:** 1 Internal Medicine, Emergency Medicine Section, United Arab Emirates University, College of Medicine and Health Sciences, Al Ain, ARE; 2 Emergency Medicine, Mersin City Education and Research Hospital, Mersin, TUR; 3 Surgery, United Arab Emirates University, Al Ain, ARE

**Keywords:** efast, medical student, rush, ultrasound, undergraduate, training, pocus, emergency medicine

## Abstract

Aim

This study evaluated the effects of three-hour instructor-led training courses in the Extended Focused Assessment Sonography for Trauma (EFAST) and Rapid Ultrasound in Shock and Hypotension (RUSH) protocols on knowledge gain and retention by final-year medical students.

Methods

This prospective study evaluated 79 final year medical students participating in an emergency medicine (EM) clerkship during the 2017-2018 academic year. None of the included students had prior formal ultrasound training or hands-on experience. All students participated in three-hour training courses on the EFAST and RUSH protocols, with training on each protocol involving one hour of didactic training and two hours of practical training. Knowledge improvement was measured by testing before and after each course, and knowledge retention was evaluated on a final clerkship multiple choice question (MCQ) examination.

Results

Median scores were significantly higher after rather than before both the EFAST (15; range, 12-19 vs. 7; range, 2-18; p < 0.0001) and RUSH (16; range, 6-20 vs. 6; range, 1-13; p < 0.0001) courses. EFAST knowledge was significantly higher than RUSH knowledge before (p = 0.04) but not after (p = 0.82) taking the respective course. The RUSH score was significantly lower than the EFAST score on the final clerkship MCQ examination (p < 0.0001).

Conclusions

Three hours of instructor-led ultrasound training given during an EM clerkship significantly increased knowledge of both the EFAST and RUSH protocols. Knowledge retention after two weeks was higher for the EFAST than the RUSH protocol. A longer period of RUSH training may improve the retention of knowledge.

## Introduction

Point-of-care ultrasound (POCUS) is regarded as important [1-4], with increasing numbers of medical schools integrating ultrasound training into their undergraduate curricula [5-7]. Ultrasound training, once only taught in residency programs or sporadically to medical students, is now included in the teaching of undergraduate students [7-10]. However, despite the consensus that ultrasound training should be part of undergraduate training, no agreements have been reached on the length of time required for training or on its content and delivery methods [5-6,11-12]. Ultrasound training during the early years of undergraduate medical education is useful for teaching basic sciences like anatomy. In later years, however, obtaining and interpreting ultrasound images becomes more important [5]. Teaching POCUS to novice learners improves their diagnostic skills, confidence, and clinical decision-making when dealing with critically ill patients [13-14]. These findings indicate that clinical clerkships should include the teaching of POCUS to medical students in different domains, including cardiology, gynecology, and critical care.

Although emergency medicine (EM) pioneered the integration of ultrasound into clerkship curricula [10,15-16], most EM clerkship curricula include little structured POCUS training [17]. Time constraints limit the integration of ultrasound training into undergraduate medical education [5,18]. This limitation has been overcome by the effective implementation of ultrasound teaching into training courses [13,17,19-20]. Our recent study showed that senior medical students can effectively learn and implement the Extended Focused Assessment Sonography for Trauma (EFAST) protocol after three hours of instructor-led training [10]. To our knowledge, however, no study to date has assessed the effectiveness of incorporating Rapid Ultrasound in Shock and Hypotension (RUSH) training into EM clerkships. The RUSH examination includes three components: the pump, tank, and pipes [21]. The pump evaluates the heart, the tank evaluates volume status and leakage, and the pipe evaluates the aorta and deep veins. RUSH considerably needs more advanced skills as compared with EFAST. However, implementing the RUSH protocol into our curriculum of the sixth-year medical students was acceptable. Our senior students are trained to make proper decision-making in shocked patients. Furthermore, our junior medical students in the fifth year are exposed to POCUS in real life during their junior surgical clerkship. This is mainly an extra-curricular activity that prepares them for more advanced decision-making skills, such as RUSH, in the sixth year [22].

The present study evaluated the effects of three-hour instructor-led training courses in the EFAST and RUSH protocols on knowledge gain and retention by final year medical students.

## Materials and methods

Ethical approval

The protocol of this prospective study was reviewed and approved by the Research and Graduate Studies Ethics Committee (Reference No: ERS-2017-5643) of our institution. All students provided written informed consent to participate.

Study setting and participants

The prospective study evaluated final-year medical students participating in an EM clerkship during the 2017-2018 academic year [23]. The clerkship trained five groups of 14 to 17 students each during the academic year. A total of 79 medical students with no prior formal ultrasound training or hands-on experience were trained on the EFAST and RUSH protocols, both in the classroom and in a skills laboratory. Two Siemens Acuson P300 portable ultrasound machines (Siemens, Munich, Germany) were available for each practical session, with each practical session taught by two to three instructors. Students were tested before and after each course by online tests via Google Documents. The final clerkship multiple-choice questions (MCQ) examination was administered by the college assessment management system in a computer-equipped examination hall. A standard-setting exercise using the modified Angoff method showed that the passing grades on the final MCQ examination for EFAST and RUSH-related questions were each 71%.

Course design

**Figure 1:**
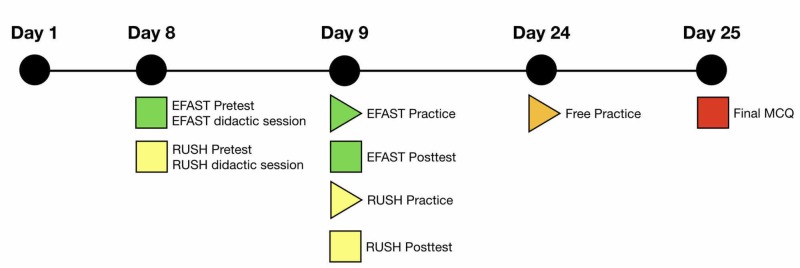
Time frame of teaching and assessment sessions for the EFAST and RUSH protocols Time frame of teaching and assessment sessions for the EFAST and RUSH protocols as part of the four-week emergency medicine clerkship during the 2017-2018 academic year. Abbreviations: EFAST, Extended Focused Assessment Sonography for Trauma; MCQ, multiple-choice question; RUSH, Rapid Ultrasound in Shock and Hypotension

Before each course, the students were administered separate 20 multiple-choice question (MCQ) tests on theoretical knowledge, practical applications, and image interpretation of the EFAST and RUSH protocols. A single tutor with 15 years of experience in POCUS presented a one-hour didactic lecture on each protocol. The two lectures included basic physics, knobology, artifacts, technique, windows, anatomical structures, pathologies, and image interpretation. Online text and video links were provided for further study at the end of the lectures (Clerkship Day 8, Figure 1). Table 1 presents the instructional design for procedure education used during the didactic and practical sessions [23-24].

**Table 1:** Gagne’s nine-step instructional design for teaching

Steps	Explanation	Comment
1	Gaining attention	Steps 1–4 were performed in the classroom during the one-hour didactic lectures.
2	Informing the learner of the objective	
3	Stimulating recall of prior knowledge	
4	Presenting information	
5	Providing guidance	Steps 5–8 were performed during the practical sessions.
6	Eliciting performance	
7	Providing feedback	
8	Assessing performance	
9	Enhancing retention and transfer	Online text/video materials were provided, and online discussion boards were used to discuss relevant cases.

On the day following each lecture, the students participated in a two-hour practical session on human models for each protocol (Clerkship Day 9, Figure 1). During each practical session, each student practiced for 15 to 30 minutes on human models under the guidance of instructors with direct feedback. All students observed their peers’ performances. At the end of each course, the students were administered the same 20 MCQ tests for each protocol (Clerkship Day 9, Figure 1).

An optional two-hour self-practice session without instructor participation was made available to each student during the last week of the rotation (Clerkship Day 24, Figure 1). The final clerkship MCQ examination, which consisted of 100 questions, included five MCQs on each protocol (Clerkship Day 25, Figure 1).

Data analysis

Because of the small number of student groups, nonparametric statistical methods were used for comparisons and correlations between groups. Results on MCQ tests before and after each course were compared using the Wilcoxon signed-rank test. Correlations between continuous variables were evaluated by Spearman's rank correlation tests. All data were analyzed using SPSS Statistics for Windows, Version 25.0. (IBM Corp, Armonk, NY), with p-values < 0.05 defined as statistically significant.

## Results

A total of 79 students were trained. Five students who trained on the EFAST protocol and nine students who trained on the RUSH protocol were excluded because they did not take tests before and/or after training.

**Figure 2:**
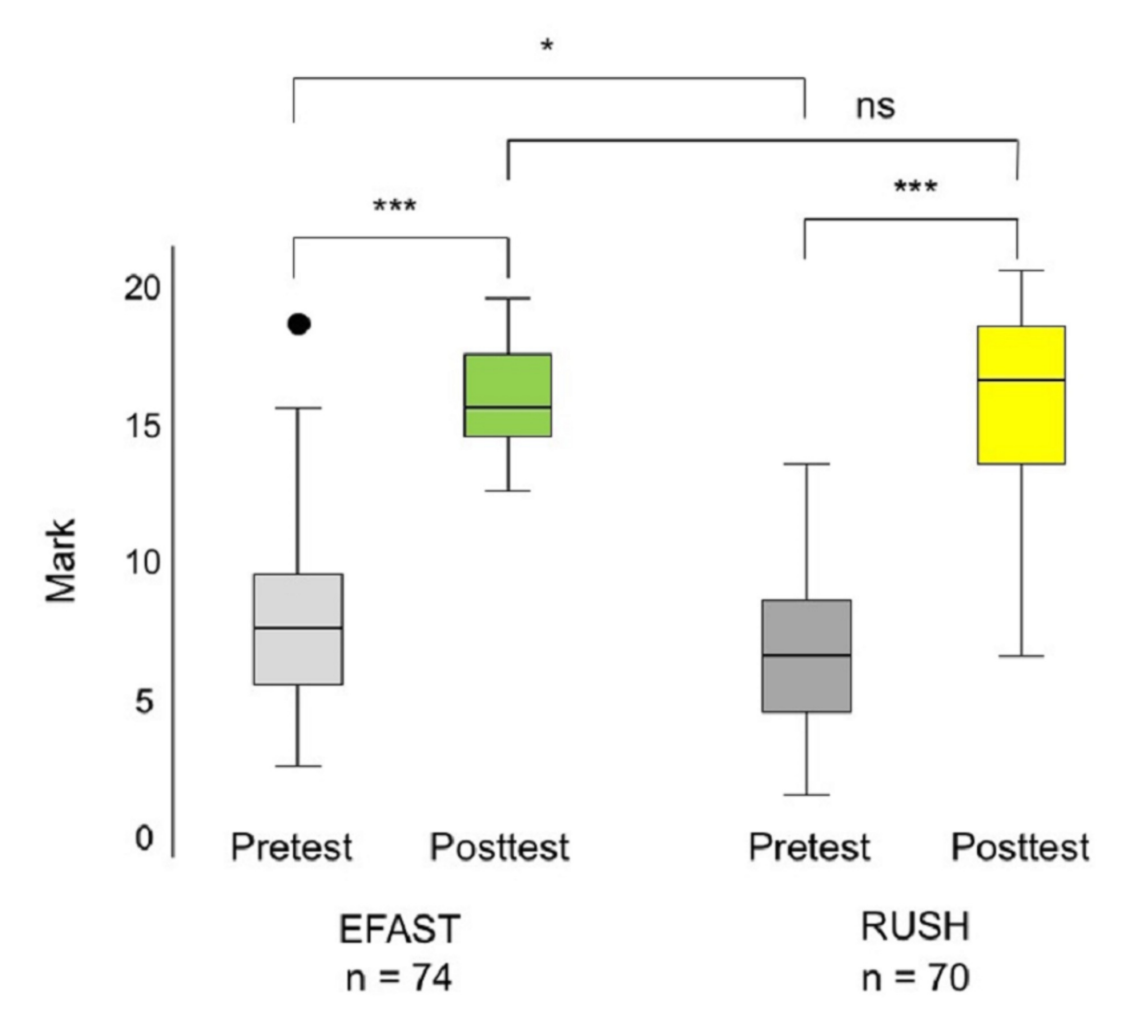
Box-and-whiskers plot of test scores of subjects before and after the EFAST and RUSH training sessions Each box shows the IQR (from the 25th to the 75th percentile). The horizontal line within each box represents the median, with black dots representing outliers. Abbreviations: EFAST, Extended Focused Assessment Sonography for Trauma; IQR, interquartile range; ns, not significant; RUSH, Rapid Ultrasound in Shock and Hypotension. *p < 0.05, ***p < 0.0001 by Wilcoxon signed-rank tests

Figure 2 shows the scores before and after the training sessions on the EFAST and RUSH protocols. Median scores were significantly higher after than before both the EFAST (15; range, 12-19 vs. 7; range, 2-18; p < 0.0001) and RUSH (16; range, 6-20 vs. 6; range, 1-13; p < 0.0001) courses. RUSH knowledge was significantly lower than EFAST knowledge before (p = 0.04), not after (p = 0.82) taking the respective course. 

**Figure 3:**
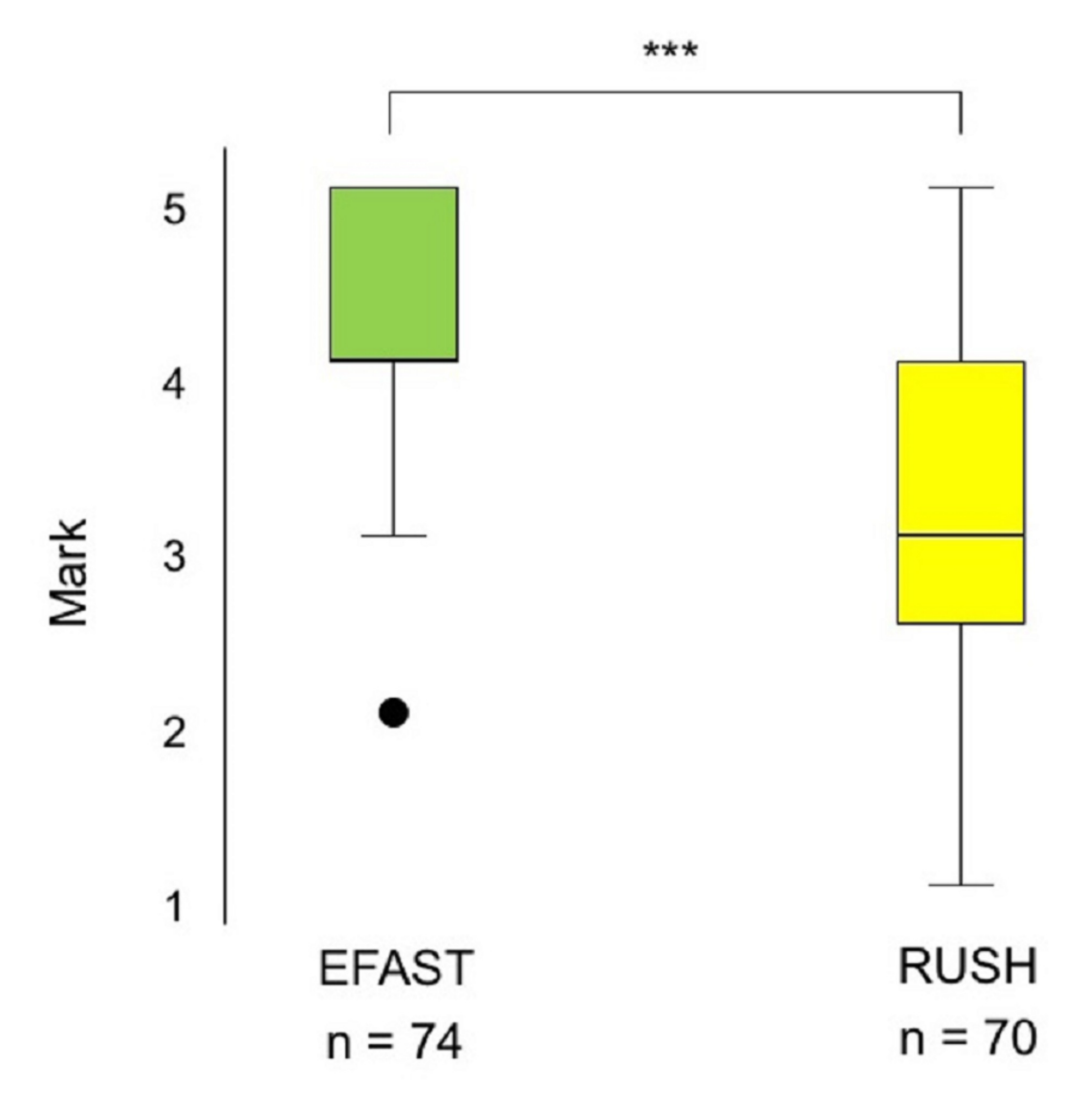
Box-and-whiskers plot of final clerkship MCQ scores on the EFAST and RUSH protocols Each box shows the IQR (from the 25th to the 75th percentile). The horizontal line within each box represents the median, with black dots representing outliers. Abbreviations: EFAST, Extended Focused Assessment Sonography for Trauma; IQR, interquartile range; MCQ, multiple-choice question; RUSH, Rapid Ultrasound in Shock and Hypotension. *** p < 0.0001 by the Wilcoxon signed-rank test.

Scores on the tests following EFAST training correlated significantly with scores on the final clerkship MCQ examination on the EFAST protocol (p <0.0001, Spearman's rank correlation rho: 0.39), with 78.5% of the students having a passing grade on the latter examination. Scores on the tests following RUSH training also correlated significantly with scores on the final clerkship MCQ examination on the RUSH protocol (p = 0.001, Spearman's rank correlation rho: 0.37), with 46.8% of the students having a passing grade on the latter examination. Median scores on the final MCQ examination were significantly lower for the RUSH than for the EFAST protocol (3; range, 1-5 vs. 4; range, 2-5; p < 0.0001; Figure 3).

## Discussion

The current study demonstrated that three-hour instructor-led training courses on the EFAST and RUSH protocols significantly increased knowledge of these protocols among final year medical students. Knowledge retention two weeks later was higher for the EFAST than the RUSH protocol.

Time restriction is a major factor limiting the integration of ultrasound training into undergraduate curricula [5,18] although training on several POCUS applications during EM clerkships remains possible. After two hours of ultrasound instruction on the FAST protocol coupled with vascular access and a supervised dedicated clinical ultrasound shift, final-year medical students performed similarly to EM residents on an MCQ examination [17]. A one-hour educational intervention was found to provide a significant increase in knowledge of POCUS [20].

Less time was needed to train on the EFAST than on the RUSH protocol, with training for one hour on EFAST effectively improving both performance and interpretation [19]. We recently reported that hands-on performance was acceptable in 88% of medical students after five hours of training, including one hour of didactic training, two hours of practical training with an instructor, and two hours of free practice [10]. The current study found that only three hours of instructor-led EFAST training was sufficient to increase knowledge significantly.

The current study also evaluated three hours of instructor-led RUSH training, finding that knowledge of this protocol increased significantly during the short term but declined within two weeks. Only 47% of undergraduate students passed the RUSH component of the final clerkship MCQ examination, compared with 79% who passed the EFAST component of this examination.

There may be several reasons for this discrepancy. First, RUSH is a longer and more complex protocol than EFAST, whereas equal time was allocated for training in both protocols. The learning load of the RUSH protocol is greater and more demanding [25]. Second, the medical students were clinically exposed more to the EFAST than the RUSH protocol [26]. Third, the EFAST examination is a component of the RUSH protocol. Retention of knowledge of the RUSH protocol may be improved by a longer period of training, refresher courses, or more clinical exposure to this protocol. Expansion of the time allocated for training on the RUSH protocol during EM clerkships is dependent on the other objectives of this curriculum.

Limitations of our study

The present study had certain limitations. First, it was a single-center study, limiting its generalizability to a broader population and other medical schools. Second, the sample size was relatively small because we included only students during one academic year. Third, all students participating in the clerkship were not included in the final analyses because some were not tested before and/or after training. Fourth, some students may have been previously exposed to either or both protocols during the first week of the rotation. Although our institution does not include formal ultrasound training in its undergraduate curriculum, some of these students may have been exposed to these protocols in other clerkships such as general surgery and internal medicine. Fifth, we used the same sets of questions in the tests administered before and after each course but different questions during the final clerkship MCQ examination. Sixth, appropriate long-term retention measurement can be challenging because it is affected by different uncontrolled factors like the student’s self-esteem of learning, learning opportunities, clinical exposure to procedures, and attending optional other POCUS courses.

## Conclusions

The present study showed that three hours of instructor-led ultrasound training given during an EM clerkship significantly increased short-term knowledge of both EFAST and RUSH protocols. Knowledge retention after two weeks was higher for the EFAST than the RUSH protocol. A longer period of RUSH training may improve knowledge retention.
